# Spatiotemporal Dynamics of Micropropagules in Seawater During the 2020 Green Tide Outbreak in the Southern Yellow Sea

**DOI:** 10.3390/biology15070591

**Published:** 2026-04-07

**Authors:** Lihua Xia, Yutao Qin, Huanhong Ji, Jiaxing Cao, Xiaobo Wang, Yuhan Zhang, Jinlin Liu

**Affiliations:** 1 Key Laboratory of Marine Ecological Monitoring and Restoration Technologies, East China Sea Ecological Center, Ministry of Natural Resources, Shanghai 201206, China; xialihua@ecs.mnr.gov.cn (L.X.); jiaxingcao910@gmail.com (J.C.); oucwangxb@163.com (X.W.); zhangyh266@foxmail.com (Y.Z.); 2 Project Management Office of China National Scientific Seafloor Observatory, Tongji University, Shanghai 200092, China

**Keywords:** macroalgal bloom, the Yellow Sea, *Ulva* Linnaeus, 1753, harmful algae, microscopic propagules

## Abstract

Large-scale green tides dominated by *Ulva* species have recurred annually in the Southern Yellow Sea for nearly two decades, yet early detection remains challenging due to the patchy distribution of incipient floating macroalgae. This study investigated the spatiotemporal dynamics of *Ulva* micropropagules during the 2020 outbreak using a systematic cultivation assay. Seawater samples were collected from 23 stations across the Subei Shoal and adjacent waters in April, May, and July, and incubated under controlled laboratory conditions to enumerate *Ulva* germling densities. Results revealed that *Ulva* micropropagule abundance peaked in April, with high-density foci concentrated in the Subei Shoal region—particularly in aquaculture areas of *Neopyropia* J. Brodie & L.-E. Yang, 2020—confirming this zone as one of the important sources. Abundance declined progressively through May and July as macroalgae drifted northward under wind and current forcing. This method effectively identified putative source regions and reconstructed initial dispersal patterns prior to satellite-detectable macroalgal aggregation. These findings demonstrate that *Ulva* micropropagule monitoring provides a cost-effective, sensitive tool for early warning and *Ulva* source tracking, offering finer-scale propagule distribution data to inform precision management strategies for mitigating green tide impacts on coastal marine ecosystems. Future research should expand investigations into *Ulva* micropropagule dynamics to elucidate their mechanistic processes and ecological significance in green tide initiation and development.

## Simple Summary

Over the past 19 years, green tides dominated by the genus *Ulva* Linnaeus, 1753, have recurred in the Southern Yellow Sea, yet early detection remains constrained by the patchy and highly mobile nature of incipient floating macroalgae under wind and current forcing. Here, we resolved the 2020 propagule landscape across the Subei Shoal and adjacent offshore waters with a micropropagule cultivation assay: seawater samples were incubated under controlled conditions and emerging germlings enumerated. The procedure reliably identified high-density foci and putative source regions, thereby reconstructing the bloom’s initial dispersal pattern. This cost-effective protocol enhances early-warning and source-tracking capacities, supporting precision management of the regional marine ecosystem.

## 1. Introduction

Large-scale green tides have bloomed in the Southern Yellow Sea of China for nineteen consecutive years; with 2019 as an example, the maximum coverage area of the green tide was 508 km^2^, the maximum distribution area was 55,699 km^2^, and the green tide persisted on the sea surface for 119 days [1]. Their extensive spatial coverage and prolonged duration have rendered them a significant challenge in the management of China’s coastal marine environment [2]. Although green tide disasters affect dozens of coastal nations worldwide, the Southern Yellow Sea events are among the most severe globally in both outbreak scale and frequency [3], posing significant threats to coastal tourism, mariculture, and marine ecosystems. One of the primary drivers has been identified as coastal eutrophication resulting from land-based nutrient inputs (nitrogen and phosphorus) derived from agricultural runoff, industrial discharge, and domestic sewage [4]. Additionally, the distinctive raft cultivation of *Neopyropia yezoensis* (Ueda) L.-E. Yang & J. Brodie, 2020 in Jiangsu coastal waters provides substantial substrate for epiphytic *Ulva* proliferation, serving as a critical biological vector for green tide initiation. In response, Chinese authorities have implemented pollution reduction strategies, including stringent mariculture waste-management regulations as well as integrated cross-regional green tide prevention and control measures [1,5,6,7]. Despite these measures, green tides have persisted, drawing sustained attention from scholars across oceanography, environmental science, and ecology; investigations span the origin and source tracking of green tides, their outbreak mechanisms, ecological impacts, and mitigation strategies [1,8,9,10], and have become a research hotspot in contemporary marine environmental research.

The dominant species responsible for green tide disasters primarily consists of green macroalgae belonging to the genus *Ulva* Linnaeus, 1753 [11]. This genus is classified within the family Ulvaceae, order Ulvales, class Ulvophyceae, and phylum Chlorophyta. It is widely distributed from temperate to tropical coastal waters worldwide, attaching to substrates such as rocky surfaces via basal rhizoids during its attached stage. *Ulva* exhibits an extremely high growth rate and strong environmental adaptability, enabling rapid propagation under a wide range of temperature, salinity, and nutrient conditions [12]. Notably, during growth and under stressful conditions, it releases micropropagules. Propagules of *Ulva* are defined as all biological units produced by algae of the genus *Ulva*, which possess the potential to develop into new algal thalli, including unicellular spores, gametes, and zygotes at the single-cell level, microscopic individuals at various developmental stages, and tissue fragments of macroalgae that are invisible at the macroscopic scale but retain regenerative capacity under suitable conditions [13]. Under suitable conditions, these propagules can attach to substrates and germinate into new algal thalli, serving as a critical “seed source” and initial biomass source for green tide outbreaks [14]. This characteristic also provides the biological foundation for indirect monitoring methods based on micropropagule cultivation.

Owing to advances in remote-sensing monitoring, physical oceanography, population genetics, and molecular ecology [15,16,17], the understanding of the Southern Yellow Sea green tides has deepened, thereby providing critical scientific support for prevention and regional ecological management. Current consensus identifies *Ulva prolifera* O.F. Müller, 1778 as the dominant causative species, whose massive accumulation is tightly linked to eutrophication in the Changjiang Estuary (noting that *U. prolifera* growth requires specific salinity conditions, whereas nearshore areas of the Changjiang Estuary are dominated by low-salinity diluted water less conducive to normal *U. prolifera* development)–Jiangsu coastal waters, the distinctive cultivation mode of the red alga *N. yezoensis* (on whose cultivation facilities epiphytic *Ulva* species readily proliferate, which become prone to detachment and floating during the *Neopyropia* harvest season), and the sea area’s specific hydro-meteorological conditions [18,19,20]. Satellite remote sensing and in situ surveys jointly reveal that green tide algae first appear on a small scale in the Subei Shoal during April–May, thereafter rapidly drifting northward under the influence of winds and surface currents; by June–July, algal aggregations exceeding tens of thousands of square kilometres can form south of the Shandong Peninsula [21]. This process markedly alters the marine ecological environment and causes direct economic losses.

Monitoring and research on the early-stage development of Southern Yellow Sea green tides have historically faced significant challenges. During initial outbreak phases, floating *Ulva* macroalgae are patchily distributed, limited in extent, and highly dynamic; conventional ship-based sampling suffers from insufficient spatial coverage (relying on visual observation of sea surfaces for macroalgae collection, which is prone to oversight during surveys; however, microscopic genetic material or environmental DNA can persist in marine environments for some time, indirectly tracking the recent distribution of macroalgae), low temporal resolution and strong sea-state dependence, precluding systematic capture of initial distributions and dispersal trajectories. Researchers have therefore introduced and refined an indirect monitoring and source-tracking method based on micropropagule cultivation [22]. Seawater samples are systematically collected throughout the region and incubated under controlled laboratory conditions that selectively cultivate *Ulva* propagules, enabling microscopic propagules to germinate and grow into macroscopic thalli. Enumeration of *Ulva* recruits per unit water volume allows calculation of population densities of dominant *Ulva* species. This technique effectively characterizes the spatiotemporal distribution of potential green tide propagules, particularly during early stages before massive thallus aggregation [23].

Preliminary studies have demonstrated distinct spring and summer maxima of *Ulva* micropropagules in the Subei Shoal and adjacent waters, whose distribution coincides with satellite-observed initial green tide locations in the Southern Yellow Sea, thereby providing pivotal evidence for identifying origin hotspots, revealing propagule-release patterns, and tracking drift pathways [24,25,26]. Micropropagule cultivation thus serves as a critical bridge linking field observations, remote-sensing retrievals, and mechanistic investigations, deepening understanding of early-stage green tide development and establishing a foundation for a more complete understanding of the entire outbreak process.

In view of the above research context and existing methodological approaches, micropropagule cultivation has become indispensable for revealing the early-stage mechanisms and dynamics of green tides, offering unique value in identifying source regions, tracing initial drift routes, and assessing recruitment potential. To advance knowledge of the spatial dynamics of Southern Yellow Sea green tides during their bloom, the present study focuses on the 2020 outbreak cycle, implementing systematic collection and laboratory cultivation of micropropagules from seawater. A station network spanning the Subei Shoal to key waters north of Jiangsu Province was deployed, and sequential sampling was conducted during pivotal phases of green tide development. Under standardised laboratory cultivation and identification protocols, quantitative analyses were performed on the spatial distribution and density dynamics of *Ulva* propagules.

The study aims to (1) clarify the spatial pattern of micropropagules during the 2020 Southern Yellow Sea green tide outbreak, identifying high-density aggregation zones and potential core source areas; and (2) preliminarily infer the early drift pathways of green tide algae. The results will provide detailed scientific evidence, based on microscopic propagule distribution, for early warning, source tracking, and mitigation of Southern Yellow Sea green tides, thereby enhancing the precision and proactive capacity of regional marine ecosystem management and supporting national strategies for sustainable marine development.

## 2. Materials and Methods

### 2.1. Station Layout and Sample Collection

**Figure 1:**
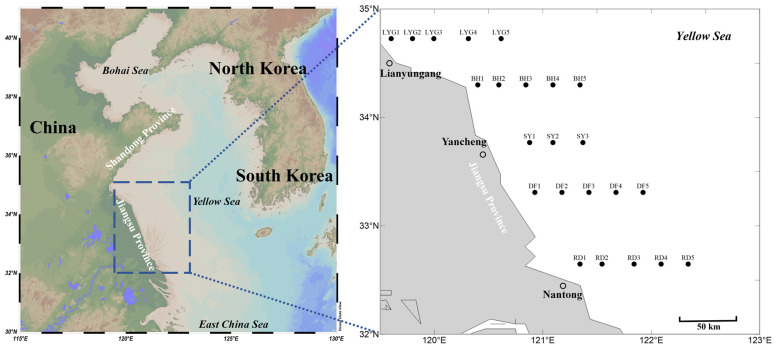
Location of the study area and station layout of the sampling sites.

Five transects were established in the Southern Yellow Sea (off the coast of Jiangsu Province), and three cruise surveys were conducted in April, May, and July 2020. A total of 23 sampling stations were set up (Figure 1), covering the offshore areas adjacent to the main coastal counties and cities of Jiangsu Province: offshore waters east of Rudong County, Nantong City (stations RD1–RD5); offshore waters east of Dafeng District, Yancheng City (stations DF1–DF5); offshore waters east of Sheyang County, Yancheng City (stations SY1–SY3; the Sheyang transect was limited to only three stations that particular year due to a combination of factors including the unique topographical and geomorphological characteristics of the area, hydrological constraints in the nearshore waters, and restrictions imposed by offshore wind farm construction during that specific year); offshore waters east of Binhai County, Yancheng City (stations BH1–BH5); and offshore waters east of Lianyungang City (stations LYG1–LYG5) (Figure 1).

At each station (Table 1), surface, middle, and bottom seawater samples were collected with a standard water sampler. Three replicate samples (1 L each) were taken from each layer. Prior to sampling, the water depth at each station was measured using the ship-mounted echo sounder. Specifically, surface water was collected at 0.5 m below the sea surface, mid-layer water was collected at half of the local water depth (mid-depth), and bottom water was collected at 2 m above the seabed (Table 1) [27].

### 2.2. Micropropagule Cultivation

Seawater samples were stored at 4 °C in the dark and transported to the laboratory immediately. To minimize external contamination, 1 L glass beakers were soaked in 10% HCl for 10 h and rinsed thoroughly with distilled water. For each station, three replicate cultivation trials were initiated by placing 500 mL of seawater into each beaker. One millilitre of saturated GeO_2_ solution was added to suppress microalgal growth (specifically to inhibit diatoms and other microalgae with silicified cell walls, which compete with *Ulva* micropropagules for nutrients and light, can cause fouling, and may also exert allelopathic effects, thereby suppressing early-stage germination and subsequent healthy growth of *Ulva* micropropagules), and 1 mL of von Stosch’s enriched (VSE) medium was supplied as a basal nutrient source [10,22,23,26].

Cultivation was performed in an incubator at 18 °C under 80–100 μmol photons m^−2^ s^−1^ with a 12 h light–12 h dark photoperiod [10,22,23,26]. To support propagule growth, 3 mL of VSE medium was replenished every fourth day. After approximately 30 days, macroscopic thalli derived from germinated micropropagules were typically observed on the inner walls and bottoms of the beakers and were subsequently underwent morphological examination and species identification.

### 2.3. Morphological Identification and Enumeration of Micropropagules

Mature macroalgal individuals obtained through micropropagule cultivation were identified to the genus level on the basis of morphological criteria. Diagnostic features included: macroscopic thalli composed of mono- or biseriate cell layers, appearing as tubular, foliose, or filamentous forms consisting of two rows of cells; attached or free-floating life habit; unbranched or diversely branched architecture; uninucleate cells containing a single, typical cup-shaped or laminate chloroplast with one to several pyrenoids; and a basal holdfast specialized for substrate attachment [28].

On the basis of these characters, professional phycologists further discriminated specimens according to classical taxonomic systems to confirm affiliation with the genus *Ulva* [28]. The integration of macro-morphology, cellular structure, and growth traits ensured reliable and scientifically rigorous classification, enabling the total number of *Ulva* individuals derived from each station to be determined. The abundance of *Ulva* micropropagules in seawater was then calculated and expressed as inds L^−1^.

### 2.4. Data Analysis

Spatial distribution patterns of micropropagules were systematically analysed with specialized marine data processing and visualization software. Specifically, Surfer 9 (Golden Software, Golden, CO, USA) was employed to interpolate and visualize the abundance data across geographic regions, producing contour maps of micropropagule density.

Descriptive statistics were calculated using Origin 2021 (OriginLab Corporation, Northampton, MA, USA). Micropropagule abundance data from all sampling stations were averaged and expressed as the mean ± standard deviation (SD). These mean values were subsequently used as input data for spatial interpolation; spatial analysis and computation were performed using kriging algorithms to reveal overall distribution trends and regional heterogeneities.

## 3. Results

### 3.1. Distribution of Ulva Micropropagules in Seawater During April

**Figure 2:**
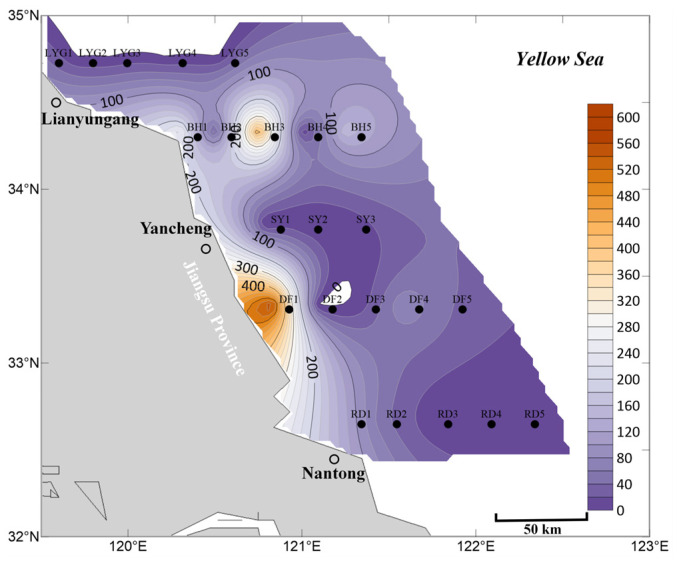
Micropropagule density in surface seawater in April 2020 (units: inds L^−1^).

In the surface seawater in April, *Ulva* micropropagules were distributed in most areas (Figure 2), indicating that direct or indirect *Ulva* release events may have occurred throughout the coastal areas of Jiangsu Province. Among them, the highest density of *Ulva* micropropagules occurred near station DF1, with the highest distribution density found from station DF1 to the nearshore area. This region is the *Neopyropia* aquaculture area in the Dongsha region of the Subei Shoal. Stations BH1, BH3, and BH5 also showed a secondary density distribution of *Ulva* micropropagules, reflecting an anomalously high distribution of *Ulva* micropropagules in the surface seawater near Binhai County (Figure 2), although the specific reasons are unknown.

Based on the data, it can be inferred that during April 2020, there was already a relatively widespread distribution of *Ulva* micropropagules in the surface seawater of the Southern Yellow Sea (Figure 2). This is strongly associated with the concentrated harvesting of *Neopyropia* in aquaculture areas during March and April, which directly or indirectly caused the release of some *Ulva* macroalgae and *Ulva* micropropagules into the Southern Yellow Sea. Alternatively, it is possible that by April, a small amount of scattered floating *Ulva* macroalgae already existed in the aforementioned offshore areas of the Southern Yellow Sea. These macroalgae continuously released *Ulva* micropropagules during their floating process, which entered the surface seawater and reached levels detectable in this study. Additionally, the spring peak in nutrient availability, whether derived from natural seasonal upwelling, deliberate fertilization by maricultural activities, or terrestrial agricultural runoff transported via riverine discharge [29,30], may have provided favorable growth conditions that triggered the germination cues for these *Ulva* micropropagules. Consequently, the release of *Ulva* micropropagules into the Southern Yellow Sea, combined with such nutrient enrichment and conducive environmental factors, introduces considerable uncertainty regarding potential green tide disasters and poses ecological risks.

**Figure 3:**
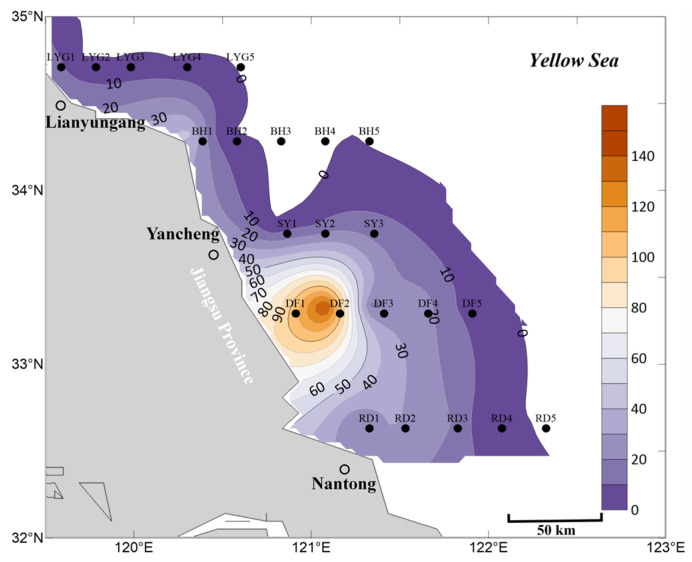
Micropropagule density in mid-layer seawater in April 2020 (units: inds L^−1^).

In the mid-layer seawater in April, the overall distribution scale and range of *Ulva* micropropagules were smaller than in the surface seawater (Figure 3). *Ulva* micropropagules were distributed from the offshore area of Nantong City to the offshore area of Lianyungang City, Jiangsu Province. The core distribution area was mainly located in the Subei Shoal and its adjacent waters, highly overlapping with *Neopyropia* aquaculture areas. The highest density of *Ulva* micropropagules was found in the Dongsha region of the Subei Shoal (mainly distributed near stations DF2 and DF1, and spatial distribution modeling results indicated a potential zone of highest density between stations DF1 and DF2) (Figure 3). This phenomenon may be related to the concentrated harvesting of *Neopyropia* and the retrieval of *Neopyropia* aquaculture rafts, human activities that typically occur in the Dongsha region in April.

Currently, most *Ulva* micropropagules in April appeared on the surface seawater (Figure 2), while a minority were already suspended in the mid-layer seawater. However, unlike the situation in surface seawater (Figure 2), the mid-layer seawater near Binhai County did not show an anomalously high distribution of *Ulva* micropropagules, although a small-scale presence was still observed at station BH1 (Figure 3). In contrast, the surface seawater near Binhai County showed an anomalously high distribution of *Ulva* micropropagules in April. This may be because the micropropagules in this area had not yet settled in large numbers into the mid-layer water, indicating that potentially existing floating *Ulva* macroalgae in this area might be in the early stages of an outbreak and had not reached the distribution range and area thresholds detectable by satellite remote sensing for green tide macroalgae. Consequently, even if small-scale floating *Ulva* macroalgae existed in this area, there were no relevant reported records for this region in April 2020. This also implies that either there might be related aquaculture activities near Binhai County, which, in the absence of green tide macroalgae prevention and cleanup efforts, led to the release of unknown quantities of floating *Ulva* macroalgae and micropropagules into the Southern Yellow Sea; or that prior to and including April, some *Ulva* macroalgae and micropropagules released from the Subei Shoal area had been transported by wind and currents to the waters near Binhai County, causing the anomalously high distribution of *Ulva* micropropagules in the surface seawater there (Figure 2). Of course, another possibility is that there might be small-scale, unknown supplemental sources of *Ulva* near the shore of Binhai County, Yancheng City, such as from tidal river mouths (estuarine channels), intertidal zones, or other habitats, which requires further investigation [30,31,32,33,34].

**Figure 4:**
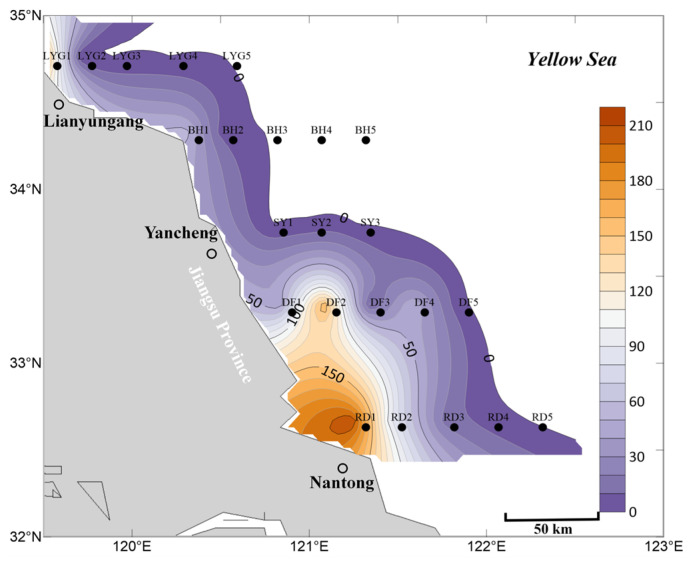
Micropropagule density in bottom seawater in April 2020 (units: inds L^−1^).

In the bottom seawater in April, *Ulva* micropropagules were also widely distributed in nearshore areas (Figure 4). Since bottom seawater is in direct contact with the benthic sediment habitat, this further reflects that the scale of the *Ulva* micropropagule “seed source” in the coastal areas of the Southern Yellow Sea in Jiangsu Province should not be underestimated.

Meanwhile, the area of highest density of *Ulva* micropropagules was located in a broad offshore area between station RD1 and station DF1 (Figure 4), most pronounced near station RD1. The reasons for this phenomenon may be as follows: First possibility (more likely): the completion time of *Neopyropia* harvesting in the waters near Rudong County might have been earlier. The abundance of *Ulva* micropropagules in the surface seawater of this area was lower than that near Dafeng District (Figure 2). This could be because *Ulva* micropropagules released earlier in the offshore area of Rudong County had already been diluted by seawater, consumed by filter feeders, died naturally, or partly settled into the bottom water–sediment interface. In contrast, the harvesting period near Dafeng District was later. Therefore, the *Ulva* micropropagules found near Dafeng District in April were mainly distributed in the surface seawater (Figure 2), but had not yet settled in large numbers into the bottom seawater habitat (Figure 4). The second possibility (less likely): as a traditional and recognized “seed source” for *Ulva* micropropagules, the Subei Shoal area, under the influence of natural hydrodynamic forces and human aquaculture activities near Rudong County during March–April, led to *Ulva* micropropagules in the sediment to be resuspended and released into the water column (Figure 4).

The secondary peak in abundance of *Ulva* micropropagules appearing in the Lianyungang City waters (near station LYG1) in April should also not be overlooked. Although the abundance and scale of *Ulva* micropropagules in the bottom seawater of this area were not comparable to those in the Subei Shoal and its adjacent waters, the distribution of *Ulva* micropropagules was detected in the surface, mid-layer, and bottom seawater at station LYG1 (Figure 2, Figure 3 and Figure 4), with the abundance order being bottom > surface > mid-layer. This indicates that *Ulva* micropropagule release events had been occurring persistently in the Haizhou Bay area prior to and including April. Some micropropagules released into the surface seawater had settled into the bottom seawater and had been settling for some time. This discovery is likely related to the existing large-scale *Neopyropia* aquaculture activities (predominantly pole-staked methods, with some floating-raft cultivation) in the Haizhou Bay area, though specific reasons require further investigation. The abundance of *Ulva* micropropagules in the bottom seawater near Binhai County in April was low, mainly appearing near station BH1 (Figure 4), with abundance also very low at station BH2.

**Figure 5:**
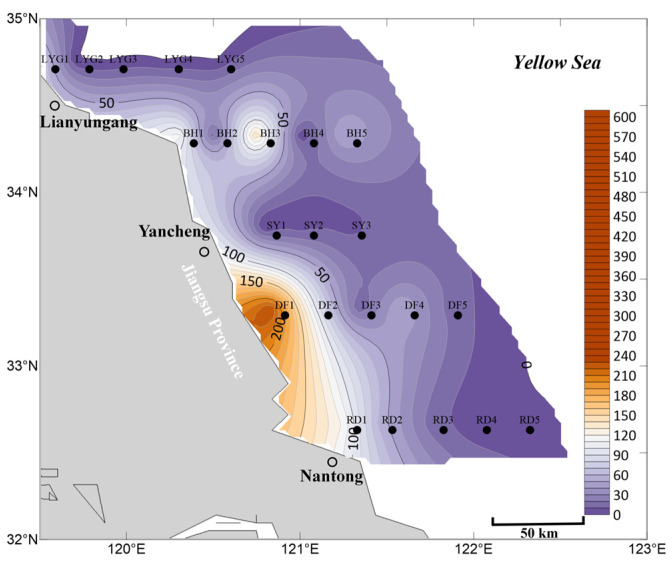
Mean abundance of micropropagules calculated as the average of surface, middle, and bottom seawater values, April 2020 (units: inds L^−1^).

Overall, based on the mean abundance of *Ulva* micropropagules in surface, mid-layer, and bottom seawater (Figure 5), the accuracy of the aforementioned findings is well supported. First, *Ulva* micropropagules were widely distributed in the offshore areas of the Southern Yellow Sea near Jiangsu Province during April (Figure 5). From the nearshore areas of Nantong City to Lianyungang City, releases of *Ulva* macroalgae and *Ulva* micropropagules into the sea area likely occurred. Second, during April, the area of highest density of *Ulva* micropropagules was located in a broad offshore area between station RD1 and station DF1 (Figure 5), which overlapped with the *Neopyropia* aquaculture areas present in the Subei Shoal region. Third, an anomalous distribution of *Ulva* micropropagules appeared along the Binhai transect in April (Figure 5), for reasons as yet unknown. Fourth, a notable abundance of *Ulva* micropropagules appeared in the Lianyungang City waters in April (Figure 5), a phenomenon that may be related to the existing *Neopyropia* aquaculture activities in the Haizhou Bay area.

### 3.2. Distribution of Ulva Micropropagules in Seawater During May

**Figure 6:**
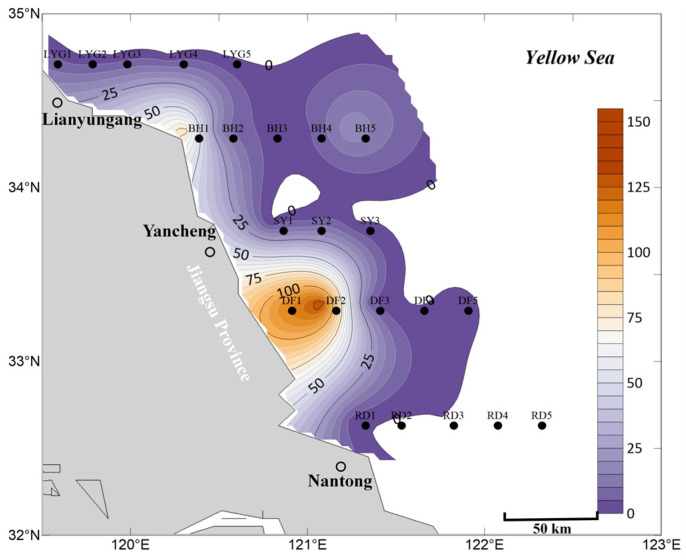
Micropropagule density in surface seawater in May 2020 (units: inds L^−1^).

**Figure 7:**
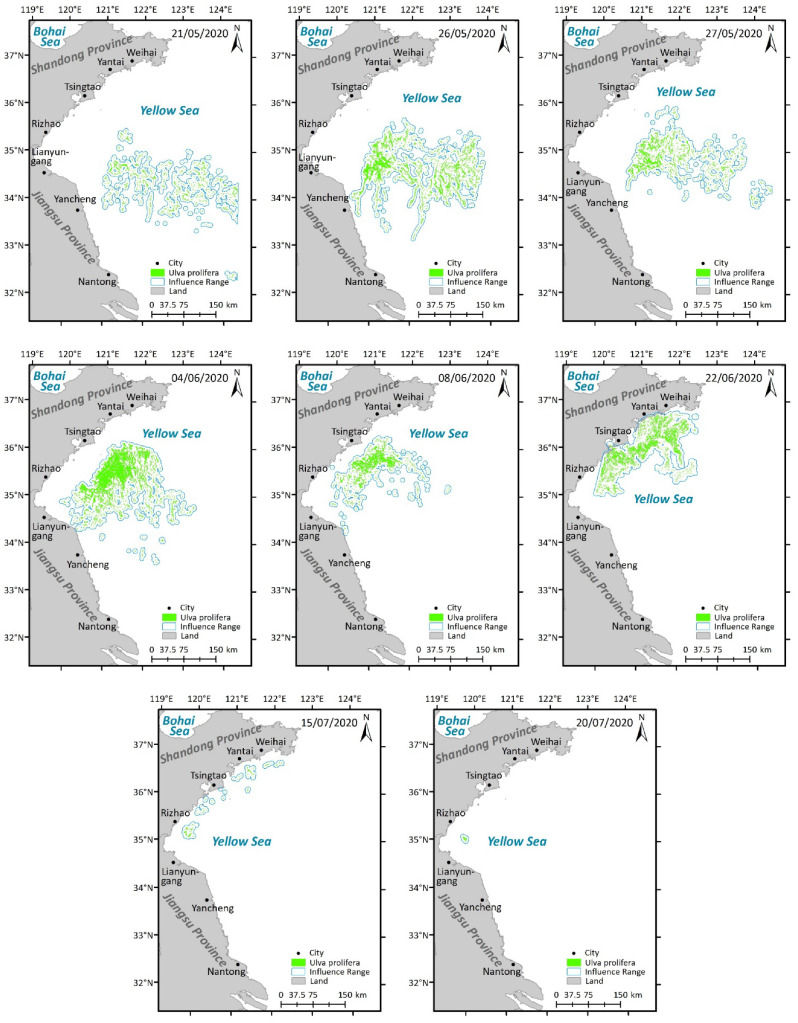
Spatio-temporal distribution of *Ulva* macroalgae in the Yellow Sea in 2020. The green areas represent *Ulva* macroalgae, observed based on data from Sentinel-2 and GaoFen-1 satellites (Cited from Wang et al. [35]; licensed under CC BY 4.0).

In the surface seawater in May, the overall abundance of *Ulva* micropropagules within the survey area was generally lower than in April (Figure 2 and Figure 6). Particularly at stations near Dafeng District, Binhai County, and Lianyungang City, the abundance of *Ulva* micropropagules in the surface seawater in May was lower than in April (Figure 2 and Figure 6). Considering that large amounts of floating *Ulva* macroalgae could be observed in the Southern Yellow Sea via satellite remote sensing starting from May 2020 (Figure 7), it is reasonable to speculate that the scale of micropropagules released by *Ulva* macroalgae in their rapid growth phase in May might be smaller than that released by the potentially existing small-scale floating *Ulva* macroalgae in their early outbreak phase in April, which were difficult to detect via satellite remote sensing. This could have led to the observed decrease in *Ulva* micropropagule abundance in the surface water near Dafeng District and Binhai County stations based on the data (Figure 2 and Figure 6). This speculation requires further verification.

Meanwhile, the area of highest density of *Ulva* micropropagules was located in a broad region of the Subei Shoal from station DF2 to the nearshore area of Dafeng District (Figure 6). However, since the scale of *Neopyropia* harvesting and raft retrieval in the Dongsha region of the Subei Shoal in May was smaller than in April, this likely resulted in a relatively smaller amount of floating *Ulva* macroalgae and *Ulva* micropropagules released from this area into the Southern Yellow Sea in May. Consequently, the observed distribution of *Ulva* micropropagules in this sea area in May was smaller than in April (Figure 2 and Figure 6). In the surface seawater in May, no large-scale distribution of *Ulva* micropropagules appeared along the Rudong transect (Figure 6), and its abundance scale was smaller than in the surface seawater in April (Figure 2).

The abundance of *Ulva* micropropagules in the surface seawater at stations along the Lianyungang transect in May was extremely low, significantly lower than in April for this region (Figure 2 and Figure 6). A large amount of floating *Ulva* macroalgae observable via satellite remote sensing already existed near station BH5 (121°15′ E, 34°20′ N) and its vicinity in May (Figure 7). Combined with the fact that a certain abundance of *Ulva* micropropagules was indeed present at station BH5 (Figure 6), it can be concluded that these micropropagules were likely released by the *Ulva* macroalgae in their rapid growth phase in this area in May, although the scale of micropropagules released during the rapid growth phase may be smaller than that during the early outbreak phase (Figure 2 and Figure 6). Furthermore, the secondary peak in abundance of *Ulva* micropropagules appearing in the nearshore area of Binhai County (near station BH1) in May should not be overlooked. However, Figure 6 and Figure 7 show that no large amounts of floating *Ulva* macroalgae observable by satellite remote sensing were present at station BH1 (120°18.321′ E, 34°20′ N) or in the nearshore area of Binhai County in May. This may be, as speculated in Section 3.1, due to the possible existence of unknown small-scale supplemental sources of *Ulva* in this area, or special hydrodynamic conditions leading to the long-term retention of floating *Ulva* macroalgae in the nearshore area of Binhai County.

**Figure 8:**
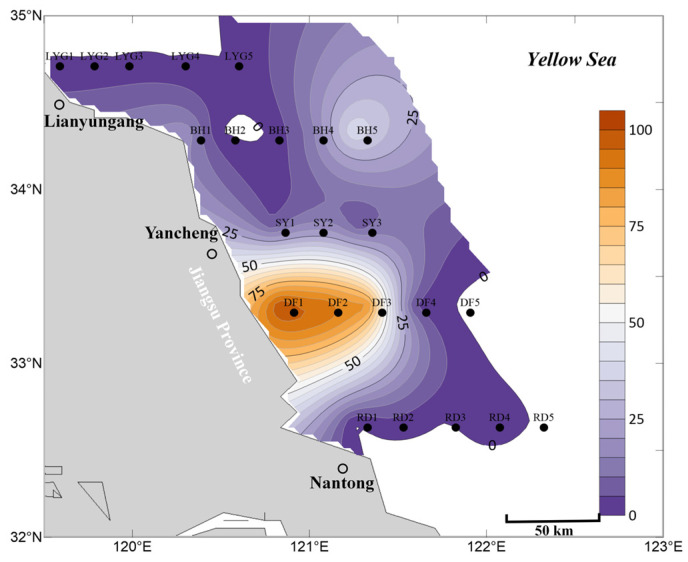
Micropropagule density in mid-layer seawater in May 2020 (units: inds L^−1^).

In the mid-layer seawater in May, the overall distribution scale of *Ulva* micropropagules was smaller than in April (Figure 3 and Figure 8), which may be related to the smaller scale of *Neopyropia* harvesting and raft retrieval in the Subei Shoal area in May compared to April. Meanwhile, the area of highest density of *Ulva* micropropagules in the mid-layer seawater in May was located in a broad region from station DF3 to the nearshore area of Dafeng District (Figure 8). This presents two major scientifically meaningful interpretations: First, some *Ulva* micropropagules gradually sink from the surface water to the mid-layer. As an important “seed source” for green tide outbreaks, the Subei Shoal area had already released a large number of *Ulva* micropropagules before floating *Ulva* macroalgae drifted away under the influence of wind and currents. Simultaneously, fragmentation of *Ulva* macroalgae induced by physical friction between aquaculture installations during *Neopyropia* growth, among other factors, or during the harvesting process, releases considerable quantities of micropropagules. Therefore, the accumulated *Ulva* micropropagules in this area were detectable in the mid-layer seawater in May (Figure 8). Second, the Subei Shoal area exhibits relatively strong hydrodynamics. Under the influence of hydrodynamics promoting diffusion, the *Ulva* micropropagules present along the Dafeng transect were gradually diluted and diffused to slightly more distant waters.

Currently, most *Ulva* micropropagules in May appeared in the surface seawater (Figure 6), while a portion had already settled and were suspended in the mid-layer seawater (Figure 8). In the mid-layer seawater in May, the scale of *Ulva* micropropagules along the Rudong transect was extremely low (Figure 8), and its abundance scale was smaller than the corresponding scale in the mid-layer seawater in April (Figure 3). Unlike the situation in surface seawater (Figure 6), the mid-layer seawater near Binhai County did not show an anomalously high distribution of *Ulva* micropropagules (Figure 8), although a small-scale presence was still observed at station BH1, which is consistent with our findings in this area in April. A large amount of floating *Ulva* macroalgae already existed near station BH5 and its vicinity in May (Figure 7). Combined with the fact that a certain abundance of *Ulva* micropropagules was indeed present in the mid-layer seawater at stations BH5 and BH4 (Figure 8), it can be concluded that these micropropagules were likely released by the *Ulva* macroalgae in their rapid growth phase in this area, with a portion of these *Ulva* micropropagules having settled and suspended in the mid-layer seawater.

**Figure 9:**
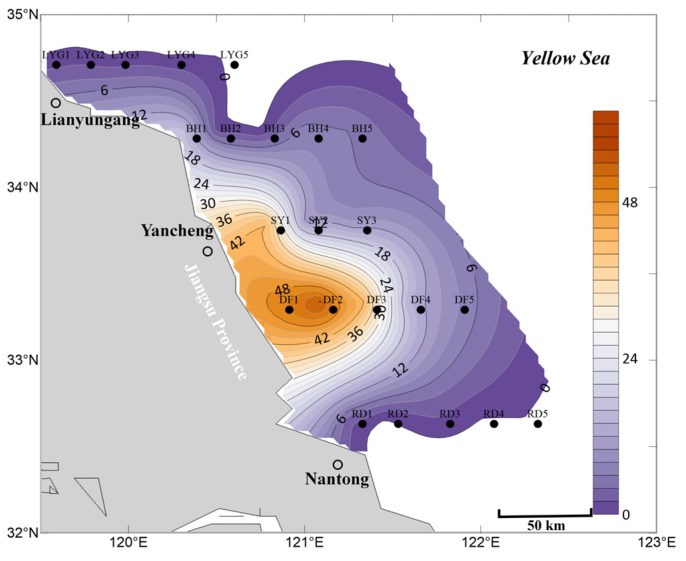
Micropropagule density in bottom seawater in May 2020 (units: inds L^−1^).

In the bottom seawater in May, *Ulva* micropropagules were also distributed in nearshore and offshore areas (Figure 9). The area of highest density of *Ulva* micropropagules was located in a broad region from station DF3 to the nearshore area of Dafeng District (Figure 9), most pronounced near station DF2. This situation is similar to that in the mid-layer seawater in May, with a slight difference: a certain abundance of *Ulva* micropropagules appeared at stations near Sheyang County (especially near station SY1), although their abundances were all lower than at stations DF1, DF2, and DF3. This phenomenon should not be underestimated. The reasons may be as follows: First possibility, the Subei Shoal area exhibits relatively strong hydrodynamics, and under the influence of coastal currents promoting diffusion, the *Ulva* micropropagules present in the Subei Shoal area were gradually diluted and diffused to slightly more distant waters, reaching the area near Sheyang County to the north by May. Second possibility, by May, a significant amount of floating *Ulva* macroalgae might have already existed on the sea surface near station SY1 (120°47.073′ E, 33°47.808′ N). In reality, this was the case, as floating *Ulva* macroalgae detectable by satellite remote sensing were objectively present near this location (120°47.073′ E, 33°47.808′ N) in the Southern Yellow Sea in May (Figure 7).

A small number of *Ulva* micropropagules were present in the bottom seawater at stations BH5 and BH4 (Figure 9). Since a large amount of floating *Ulva* macroalgae already existed near station BH5 and its vicinity in May (Figure 7), it can be concluded that these micropropagules may have been released by the *Ulva* macroalgae in their rapid growth phase in this area, with some of the micropropagules released into the surface seawater having gradually settled into the bottom seawater.

Different from the situation in April, the abundance of *Ulva* micropropagules in the bottom seawater near Rudong County in May was extremely low (Figure 9). March-April is an important period for the completion of *Neopyropia* harvesting near Rudong County. By the time of water sample collection for this study in May, more than a month had passed since the release of *Ulva* macroalgae and *Ulva* micropropagules from this area. April-May is an important period for the completion of *Neopyropia* harvesting near Dafeng District. By the time of water sample collection for this study in May, less than a month had passed since the release of *Ulva* macroalgae and *Ulva* micropropagules from this area. Therefore, a certain abundance of *Ulva* micropropagules still existed in the bottom seawater near Dafeng District in May (Figure 9).

**Figure 10:**
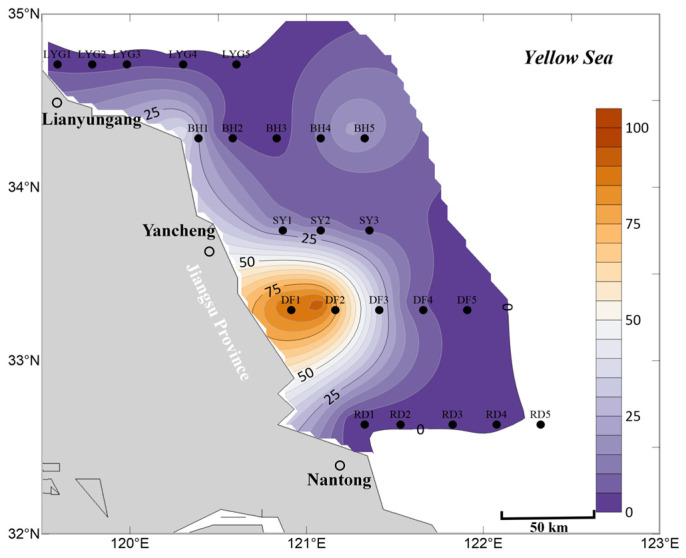
Mean abundance of micropropagules calculated as the average of surface, middle, and bottom seawater values, May 2020 (units: inds L^−1^).

Overall, based on the mean abundance of *Ulva* micropropagules in surface, mid-layer, and bottom seawater (Figure 10), the accuracy of the aforementioned findings is well supported. First, distribution on a certain scale of *Ulva* micropropagules still existed in the offshore areas of the Southern Yellow Sea in Jiangsu Province during May (Figure 10), with *Ulva* micropropagules continuously diffusing in the sea area. Second, during May, the area of highest density of *Ulva* micropropagules was located in a broad sea area from station DF2 to the vicinity of Dafeng District (Figure 10), which overlapped with the *Neopyropia* aquaculture areas present in the Subei Shoal region. Third, the scale of *Ulva* micropropagules in the seawater of the study area in May was smaller than in April (Figure 5 and Figure 10). Fourth, an anomalous distribution of *Ulva* micropropagules still appeared along the Binhai transect in May (Figure 10), for reasons as yet unknown.

### 3.3. Distribution of Ulva Micropropagules in Seawater During July

**Figure 11:**
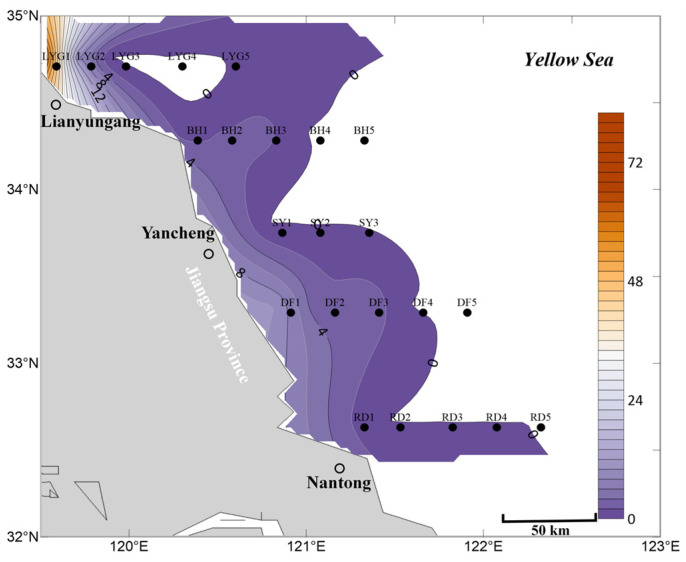
Micropropagule density in surface seawater in July 2020 (units: inds L^−1^).

In the surface seawater in July, *Ulva* micropropagules were distributed in some areas within the survey region (Figure 11). The overall distribution scale and abundance were lower than in April and May (Figure 2, Figure 6 and Figure 11). On one hand, in July, there were small quantities of *Ulva* micropropagules in the surface seawater of most nearshore areas in Jiangsu Province and the radial sand ridge area of the Subei Shoal. Their distribution scale and abundance were lower than in April and May (Figure 2, Figure 6 and Figure 11), further indicating that the scale of floating *Ulva* macroalgae in this region was very limited in July 2020. Over time, aside from portions of algal material that had settled or experienced natural mortality, remaining *Ulva* macroalgae in the Subei Shoal ridge were gradually transported into open waters of the Southern Yellow Sea under wind and current forcing (Figure 7). On the other hand, a certain abundance of *Ulva* micropropagules was detectable at both stations LYG1 and LYG2 along the Lianyungang transect (Figure 11). Among the surface water samples collected in July for this study, station LYG1 had the highest *Ulva* micropropagule abundance of all stations (Figure 11). This may be because the large-scale floating *Ulva* macroalgae in the Southern Yellow Sea had drifted to the Lianyungang transect by July 2020. In line with actual observations, a certain scale of floating *Ulva* macroalgae was indeed present near station LYG1 (119°30.286′ E, 34°45.835′ N) in July 2020 (Figure 7). Furthermore, during the final disappearance stage of the green tide in 2020, the last small patch of substantial floating *Ulva* macroalgae gradually settled and vanished in the waters near Lianyungang City and Rizhao City (Figure 7).

**Figure 12:**
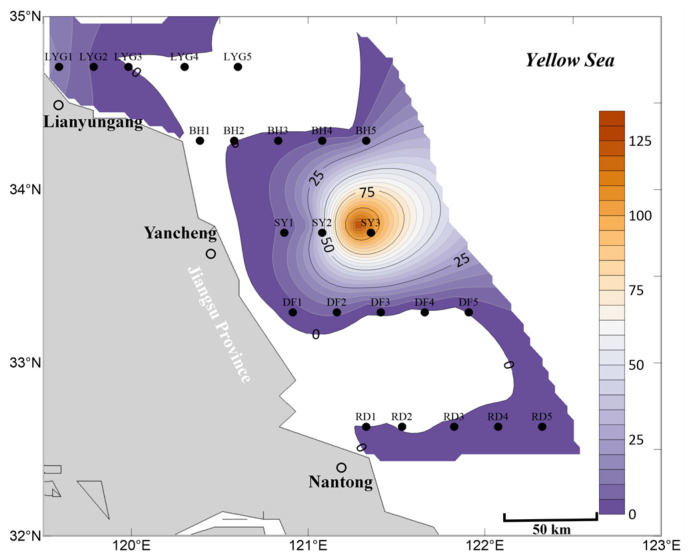
Micropropagule density in mid-layer seawater in July 2020 (units: inds L^−1^).

In the mid-layer seawater within the survey region in July, the overall distribution scale of *Ulva* micropropagules was smaller than in the mid-layer seawater in April and May (Figure 3, Figure 8 and Figure 12). On one hand, in July, no *Ulva* micropropagule distribution was detected in some areas of the mid-layer seawater in the nearshore regions of Jiangsu Province and the radial sand ridge area of the Subei Shoal. Very small amounts of *Ulva* micropropagules were detected in a few areas, such as the offshore area of Lianyungang City (Figure 12). The detection of a small number of *Ulva* micropropagules in the mid-layer seawater of the Lianyungang City offshore area is likely related to the micropropagules released by the floating *Ulva* macroalgae present there in July (Figure 7), which had gradually sunk from the surface layer to the mid-layer. An anomalous peak in *Ulva* micropropagules appeared along the Sheyang transect (especially near station SY3). It is speculated that this may be because some floating *Ulva* macroalgae in this area had entered the senescence phase and gradually settled into the mid-layer water (or there might be suspended algal filaments in this area).

**Figure 13:**
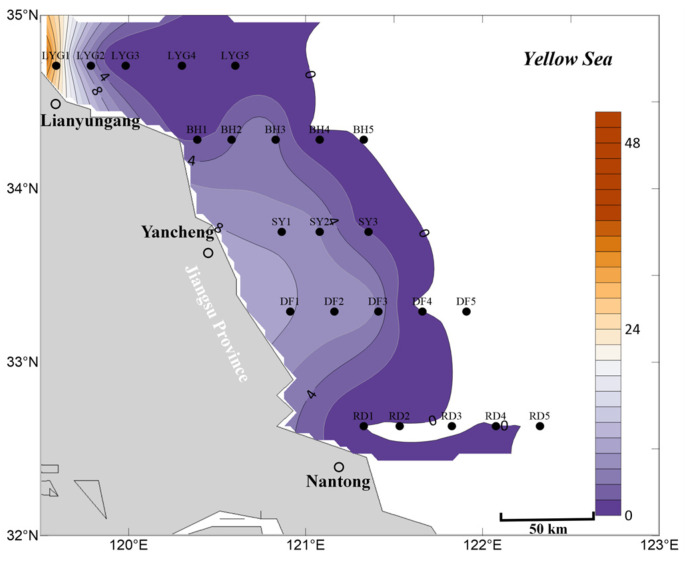
Micropropagule density in bottom seawater in July 2020 (units: inds L^−1^).

In the bottom seawater within the survey region in July, the overall distribution scale of *Ulva* micropropagules was smaller than in the bottom seawater in April and May (Figure 4, Figure 9 and Figure 13). In July, only very small amounts of *Ulva* micropropagule distribution were detected in some areas of the bottom seawater in the nearshore regions of Jiangsu Province and the radial sand ridge area of the Subei Shoal. A peak in *Ulva* micropropagule abundance appeared at the nearshore station of the Lianyungang transect, but its scale was also relatively limited. The reason for this is that a certain scale of floating *Ulva* macroalgae was indeed present in this area in July 2020 (Figure 7) and ultimately settled there. During the senescence stage, the algal bodies gradually released *Ulva* micropropagules, which settled into the bottom water layer.

**Figure 14:**
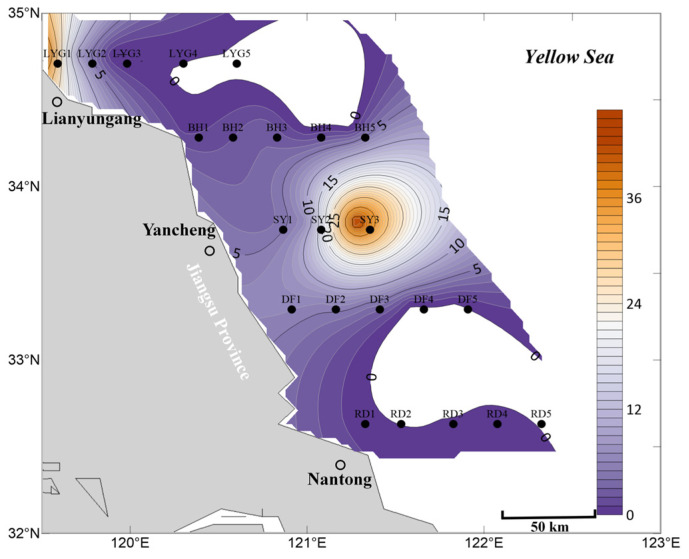
Mean abundance of micropropagules calculated as the average of surface, middle, and bottom seawater values, July 2020 (units: inds L^−1^).

Overall, based on the mean abundance of *Ulva* micropropagules in surface, mid-layer, and bottom seawater (Figure 14), the findings are consistent with the description above. In July, the abundance, distribution, and scale of *Ulva* micropropagules in the seawater of the survey area were primarily influenced by factors such as the release of *Ulva* micropropagules by floating *Ulva* macroalgae during their drifting process, and floating *Ulva* macroalgae that had entered the senescence phase, gradually settling into the seawater (or the possible presence of suspended algal filaments in the area) and releasing *Ulva* micropropagules.

## 4. Discussion

### 4.1. Significance of Micropropagule Research

The algal species composition of the Southern Yellow Sea green tide exhibits a distinct succession pattern: the early outbreak phase typically includes various *Ulva* species such as *Ulva linza* Linnaeus, 1753, *Ulva prolifera*, *Ulva compressa* Linnaeus, 1753, and *Ulva flexuosa* Wulfen, 1803; it is not until the rapid growth phase and subsequent stages that *Ulva prolifera* develops into the absolutely dominant species [36,37,38,39,40,41]. Therefore, analyzing the genus *Ulva* as a whole in scientific research contributes to a more comprehensive and objective understanding of the mechanisms behind green tide occurrences. The study of *Ulva* micropropagules is an important manifestation of this approach.

The results of this study indicate that during the early stages of the green tide (Figure 2, Figure 3, Figure 4, Figure 5, Figure 6, Figure 7, Figure 8, Figure 9 and Figure 10), the high-density areas of *Ulva* micropropagules were mainly concentrated in the Subei Shoal and its adjacent waters. This region features the development of over 70 tidal sand ridges (e.g., Dongsha near Dafeng District, JiangJiasha and ZhuGensha near Rudong County), with sediments primarily derived from the paleo-Yangtze River discharge and the ancient Yellow River outflow channels [42,43]. The formation of these sand ridges originated from the southward migration of silt transported by the Yangtze River, which entered the sea near Jianggang Port during the early Holocene. Under the combined influence of the East China Sea advancing tidal wave and the Yellow Sea rotary tidal wave, a large radial tidal sand ridge group was formed on the inner shelf of the southwestern Yellow Sea [42,43]. Numerous studies have pointed out that the Southern Yellow Sea green tide primarily originates from the Subei Shoal area and is closely related to aquaculture activities in this region [44,45]. Human activities further promote the direct release of discarded *Ulva* macroalgae from aquaculture facilities [1], and also indirectly facilitate the entry of *Ulva* micropropagules into the sea area [45,46]. Based on the spatiotemporal distribution data of *Ulva* micropropagules (Figure 1, Figure 2, Figure 3, Figure 4, Figure 5, Figure 6, Figure 7, Figure 8, Figure 9, Figure 10, Figure 11, Figure 12, Figure 13 and Figure 14), this study further clarifies, from the perspective of propagule dynamics, the close ecological association and causal link between the Southern Yellow Sea green tide and the Subei Shoal area. This demonstrates the significant scientific value of researching micropropagules, warranting further in-depth related studies in this area in the future.

### 4.2. Interannual Dynamics of Ulva Micropropagules in the Southern Yellow Sea Require Attention

The year 2020 was particularly important for green tide prevention and control in the Southern Yellow Sea. Judging from the actual areal extent of green tide outbreaks, associated prevention and mitigation measures likely achieved measurable efficacy: the maximum coverage area of the green tide outbreak that year was 192 km^2^ [1], making the outbreak scale relatively small compared to previous years. However, limited by the impact of the COVID-19 pandemic, *Ulva* micropropagule surveys that could be conducted in 2020 were also relatively restricted. Seawater samples were only collected in the study area in April, May, and July of 2020. If conditions permit in the future, monthly monitoring frequency could be increased appropriately to further enhance monitoring accuracy and verify the practical effectiveness of green tide prevention and control efforts. Furthermore, this study found that before the green tide outbreak, a notable abundance of *Ulva* micropropagules existed at the offshore stations along the Lianyungang transect (Figure 4), but their scale in 2020 was far less than that in the Subei Shoal waters. It is recommended that the number of stations set up in the Haizhou Bay area be appropriately increased in subsequent studies, which would facilitate research on the release scale of *Ulva* micropropagules in this region during the aquaculture cycle.

**Figure 15:**
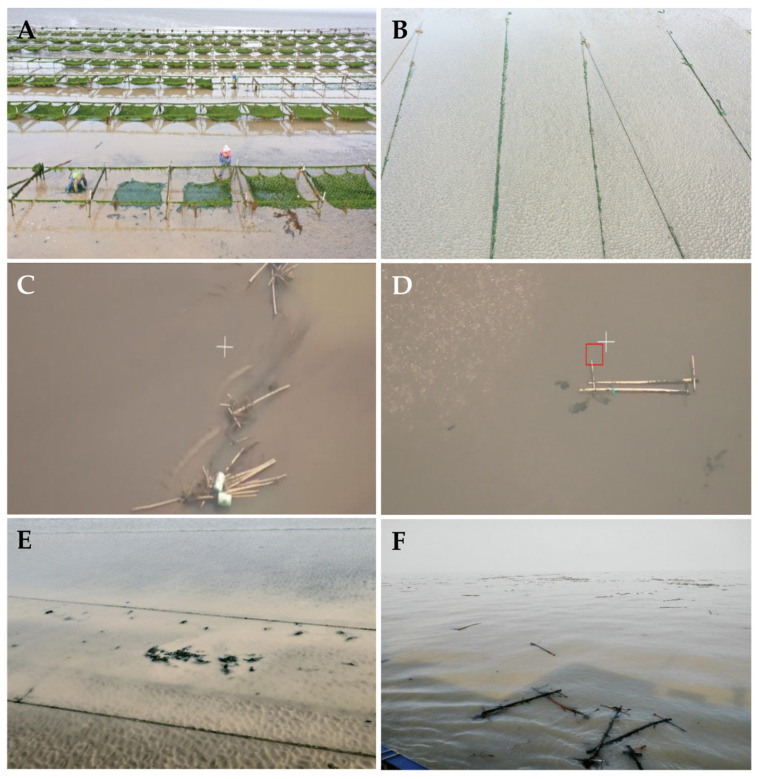
In April 2020, on the *Neopyropia* aquaculture raft facilities of local mariculture enterprises, poor growth of *Neopyropia* resulted in *Ulva* macroalgae extensively occupying the ecological niche in large quantities. During the farmers’ recycling of the raft facilities, *Ulva* macroalgae detached and drifted away in the sea area (**A**). In April 2020, during the retrieval of *Neopyropia* aquaculture raft facilities by local mariculture enterprises, raft mooring ropes were temporarily placed on the tidal flat, and *Ulva* macroalgae became detached during this process (**B**). In April 2020, local mariculture enterprises intentionally discarded raft bamboo poles on the tidal flat, with *Ulva* macroalgae attached to the poles (**C**). In April 2020, local mariculture enterprises intentionally discarded raft bamboo poles, during which process *Ulva* macroalgae detached and were left in the sea area (**D**). In April 2020, local mariculture enterprises improperly cleaned *Ulva* macroalgae attached to raft mooring ropes, which subsequently fell onto the tidal flat (**E**). In May 2020, a large number of drifting bamboo poles were discovered in the Subei Shoal waters. These poles were components of *Neopyropia* aquaculture raft facilities, with large quantities of *Ulva* macroalgae attached to them, and sporadic *Ulva* macroalgae were already floating in this area (**F**).

Simultaneously, during the process of monitoring and preventing the abandonment of discarded *Ulva* macroalgae in aquaculture areas, we observed that some aquaculture enterprises or individual farmers still engaged in various non-standard and improper aquaculture practices. These included the abandonment of facility-fouling *Ulva* macroalgae on tidal flats, excessive growth of green algae on nets, the toppling of aquaculture rafts, and the detachment and drifting of bamboo rafts (Figure 15). Following high tide, the corresponding *Ulva* macroalgae were subsequently released into the waters near the Subei Shoal. This portion of *Ulva* macroalgae, directly released into the sea, along with the indirectly formed *Ulva* micropropagules, constituted an important propagule source for the 2020 Southern Yellow Sea green tide disaster [1]. Subsequently, under the influence of marine physical, chemical, and biological factors such as localized rainfall and wind- and current-driven algal dispersal [47,48,49,50,51,52], the scale of the Yellow Sea green tide outbreak further expanded, ultimately reaching a maximum coverage area of 192 km^2^ on 29 June 2020 [1]. Currently, the Southern Yellow Sea experiences both natural outbreak years and human intervention years (where pilot green tide prevention and control trials and emergency algae removal operations were conducted in some years, including scale control of *Neopyropia* aquaculture, chemical algae removal, early-stage algae salvage, and strict nutrient input supervision) [41]. In the practical implementation of green tide prevention and control, various unexpected situations [1], varying natural and anthropogenic factors, and distinct marine environmental backgrounds emerge each year, making it challenging to interpret *Ulva* micropropagule data with sufficient clarity. From the perspective of practical green tide control objectives, reducing the scale of the *Ulva* micropropagule “seed source” in the Subei Shoal area has been a long-standing marine management goal. Therefore, it is necessary to continue conducting multi-year, multi-scale research on *Ulva* micropropagules to objectively assess the specific effects of Yellow Sea green tide prevention and control trials.

### 4.3. Close Link Between the Distribution of Ulva Micropropagules and Floating Ulva Macroalgae

**Figure 16:**
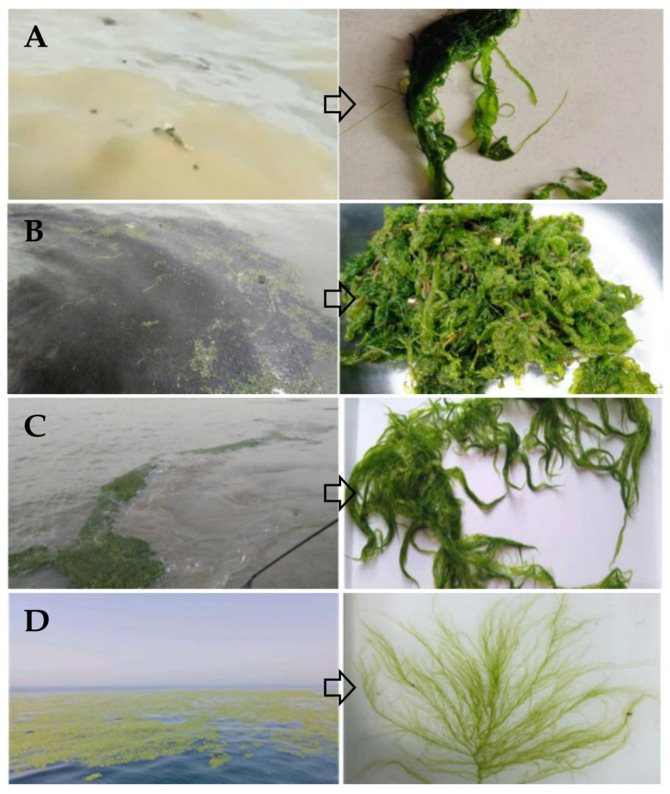
Small-scale floating *Ulva* macroalgae discovered in the waters near Taiyang Island, Rudong County, on 31 March 2020 (**A**); Small-scale floating *Ulva* macroalgae and floating *Sargassum* macroalgae discovered in the waters near Taiyang Island, Rudong County, on 4 April 2020 (**B**); Aggregated floating *Ulva* macroalgae in the rapid growth phase discovered in the waters near Dafeng District on 12 May 2020 (**C**); Large-scale floating *Ulva* macroalgae in the stable growth phase discovered in the offshore waters of Shandong Province in early June 2020 (**D**).

The outbreak process of floating green tide macroalgae in the Southern Yellow Sea can typically be divided into the early outbreak phase, rapid growth phase, stable growth phase, and senescence phase. This division is primarily based on satellite remote sensing imagery and field observation results [53,54,55,56,57]. Although there are some differences in the nomenclature of stages among different studies, often stemming from variations in terminology translation, classification criteria, study areas, and perspectives, all classification systems share the core objective of describing the scale and developmental stage of the green tide. The key lies in accurately reflecting the distribution of *Ulva* macroalgae in the sea area (Figure 16).

When the green tide scale is small (Figure 16) and difficult to effectively identify via satellite remote sensing, the discovery of floating *Ulva* macroalgae usually relies on field survey methods such as vessel-based observations (Figure 16). Research on *Ulva* micropropagules further provides an important method for monitoring this stage. Current academic research generally follows this observational pattern: when the scale of floating *Ulva* macroalgae in a sea area is large, the corresponding abundance of *Ulva* micropropagules is usually also high; similarly, when the scale of settled *Ulva* macroalgae in a sea area is large, the abundance of micropropagules also increases accordingly; furthermore, during the stage when algal fragments initially break off and enter the water body, the number of released micropropagules is also significant. *Ulva* micropropagules and *Ulva* macroalgae are closely linked. However, how to precisely quantify the functional relationship between the two remains a key issue to be further explored and addressed in future research.

### 4.4. The Nearshore Area near the Binhai Transect Requires Further Attention

**Figure 17:**
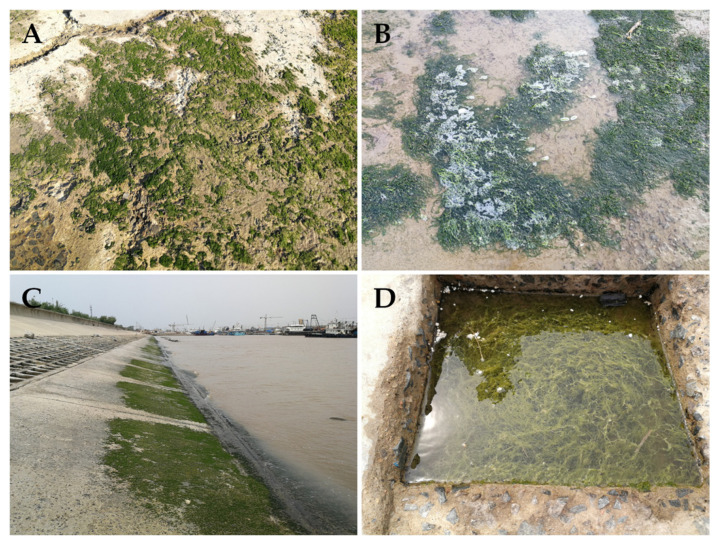
Attached *Ulva* macroalgae present in the Huaihe River seaward waterway (**A**,**B**) and attached *Ulva* macroalgae present on the seawall near Binhai Port (**C**,**D**).

This study found that during the early stage of the green tide outbreak, there was a slightly higher abundance of *Ulva* micropropagules in the nearshore area of Binhai County (e.g., Figure 6), although the specific reasons are unknown. Preliminary analysis suggests this phenomenon may be related to attached green tide macroalgae in the nearshore area of this region and floating green tide macroalgae in the river channels discharging into the sea [58,59,60,61]. As an example, on 28 May 2020, we found a certain scale of attached *Ulva* macroalgae at the sluice gate of the Huaihe River seaward waterway near Binhai County (Figure 17A,B); on 29 May 2020, we also found a large amount of attached *Ulva* macroalgae on the seawall near Binhai Port (Figure 17C,D). Whether these *Ulva* macroalgae influence the abundance of *Ulva* micropropagules in the nearshore area of Binhai County requires further investigation.

Existing research also preliminarily indicates that multiple *Ulva* species, including *U. prolifera*, *U. flexuosa*, *U. linza*, and *U. simplex*, exist in the nearshore mariculture areas and river channels discharging into the sea in Jiangsu Province, and exhibit substantial biomass. In February, *U. prolifera* was the dominant species at various sampling points [58]. A 2009 study by Pang et al. [29] suggested that *Ulva* macroalgae in the nearshore mariculture areas of Jiangsu Province are an important source of the Southern Yellow Sea green tide. At least six *Ulva* species (*Ulva prolifera*, *Ulva meridionalis* R. Horimoto & S. Shimada, 2011, *Ulva linza*, *Ulva flexuosa*, *Ulva californica* Wille, 1899, and *Ulva intestinalis* Linnaeus, 1753) were found among the attached green macroalgae on the Binhai Port seawall. In April 2022, the biomass of attached green macroalgae on the Binhai Port seawall even reached approximately 26 metric tons (wet mass) [60]. Furthermore, during scour by currents, some attached green macroalgae detach and subsequently enter the sea as free-floating algal bodies [61], posing additional ecological risks. Therefore, the specific contribution of *Ulva* macroalgae existing in various habitats to green tide outbreaks still needs further clarification [62,63].

Of course, we currently cannot rule out other factors. For instance, there is the possibility that floating *Ulva* macroalgae had already drifted from the Subei Shoal waters to the Binhai transect in May 2020, thereby causing the slightly higher abundance of *Ulva* micropropagules in the nearshore area of Binhai County (e.g., Figure 6). Overall, the *Ulva* macroalgae and *Ulva* micropropagules present in Binhai County and areas adjacent to it exhibit significant research value and potential for further study.

### 4.5. Room for Improvement in Ulva Micropropagule Research

Based on the inference from 2020 propagule data, April and earlier months likely constitute a significant release period for *Ulva* macroalgae and *Ulva* micropropagules entering the sea. As algal fragments initially enter the marine environment in a fragmented state, under the influence of variable marine environmental factors and grazing by aquatic animals, floating *Ulva* macroalgae in the early outbreak phase may release micropropagules in large quantities to adapt to the marine environment, leading to locally elevated abundances of *Ulva* micropropagules. Subsequently, the algae enter a rapid growth phase, during which their molecular regulation is primarily focused on proliferation. As the algae transition into stable growth and later senescence phases, their molecular regulation becomes increasingly dominated by reproduction [64,65,66,67,68].

Future research should incorporate controlled quantitative laboratory experiments. By designing gradient treatments of single and combined environmental factors, the actual scale of micropropagule release from different *Ulva* species at different growth stages can be analyzed. Based on experimental data, functions and response equations should be constructed to establish a quantitative model for micropropagule release from *Ulva* macroalgae in natural environments. Furthermore, investigation of the correlation between floating green tide algal biomass on the sea surface and the abundance of *Ulva* micropropagules in seawater will enable optimization of model parameters for more accurate assessment of the effectiveness of Yellow Sea green tide prevention and control measures in a given year. Concurrently, a series of gradient experiments should be conducted to explore the survival duration and dormancy state of *Ulva* micropropagules under varying conditions of water temperature, light, air temperature, humidity, and hydrostatic pressure. As micropropagules disperse in the sea, the results of such research will help quantify the potential risk of green tide disasters in target sea areas in the future.

Additionally, the survey methods used to study *Ulva* micropropagules urgently require technological innovation. Currently, some researchers have attempted to use environmental DNA (eDNA) technology to detect *Ulva* micropropagules in the Southern Yellow Sea [69]. However, this technology still has several notable limitations: Firstly, it is prone to producing false-positive results. Secondly, eDNA has a defined degradation period in the marine environment, and its signal intensity is influenced by multiple factors such as water temperature, pH, microbial activity, and hydrodynamic conditions, limiting its temporal and spatial representativeness. Thirdly, existing research indicates that eDNA technology cannot yet fully replace conventional monitoring of population or community abundance and biomass dynamics, nor can it accurately obtain genetic information crucial for conservation and management, such as genetic diversity and population structure (due to the high similarity of marker sequences among species within the genus *Ulva*, the resolution of existing eDNA metabarcoding markers, e.g., 18S V4, *rbc*L, is limited). Therefore, it is largely regarded as a supplementary tool to traditional monitoring methods [70].

Moreover, current eDNA studies related to *Ulva* often lack systematic validation through quantitative and qualitative laboratory and field experiments prior to their application in *Ulva* monitoring. For instance, the amount of DNA released by *Ulva* at different life history stages and its detectable duration have not been clarified, nor has a reliable conversion model between eDNA concentration and algal cell quantity been established. Regarding sampling strategies, further optimization is needed by integrating cutting-edge research both domestically and internationally [71], including aspects such as sampling volume, frequency, depth layers, and filter membrane material, to reduce errors and improve result comparability [72,73,74,75,76,77,78,79,80,81,82]. These shortcomings result in numerous methodological limitations and research gaps in the application of this technology to *Ulva* micropropagule detection. Future efforts should strengthen the evaluation of the accuracy, sensitivity, and standardization procedures of eDNA technology itself, particularly by combining controlled laboratory experiments with field validation to establish eDNA monitoring standards applicable to *Ulva*. Simultaneously, the potential of other emerging technologies for detection and application in this field should be actively explored.

## 5. Conclusions

This study elucidates the spatiotemporal dynamics of *Ulva* micropropagules during the 2020 green tide outbreak in the Southern Yellow Sea, revealing distinct distribution patterns across three water layers from April to July. In April, micropropagules were widely distributed throughout the surface, middle, and bottom waters, with the highest densities concentrated in the Subei Shoal and adjacent waters, particularly near *Neopyropia* aquaculture areas such as the Dongsha region, confirming these zones as critical source areas. Notably, an anomalously high distribution appeared near Binhai County, suggesting potential unknown supplemental sources or early-stage floating macroalgae that escaped satellite detection. By May, surface water abundance declined significantly compared to April, while micropropagules progressively settled into middle and bottom waters, indicating continuous diffusion and vertical migration processes; this reduction likely reflects lower release rates from macroalgae in rapid growth phases compared to early outbreak stages. In July, overall abundance and distribution scales decreased substantially as floating macroalgae drifted northward and entered senescence, with residual micropropagules detected primarily near Lianyungang waters, where the final algal mats ultimately dissipated. These findings demonstrate that micropropagule abundance peaks during early outbreak phases, and that cultivation assays effectively identify source regions and early dispersal pathways that precede satellite detectable blooms. Future research should establish quantitative relationships between micropropagule release and macroalgal biomass dynamics, and integrate molecular techniques with hydrodynamic modeling to enhance predictive monitoring and enable precision management of green tide disasters.

## Figures and Tables

**Table 1:** Surface depth, mid-layer depth, and bottom depth for seawater sampling at each station during April, May, and July 2020.

Sampling Station	Sampling Date (Year-Month)	Surface Seawater Sampling Depth/m	Mid-Layer Seawater Sampling Depth/m	Bottom Seawater Sampling Depth/m
RD1	April 2020	0.5	7	12
RD2	April 2020	0.5	11	20
RD3	April 2020	0.5	9.35	16.7
RD4	April 2020	0.5	9.5	17
RD5	April 2020	0.5	13.5	25
DF1	April 2020	0.5	12	22
DF2	April 2020	0.5	5	8
DF3	April 2020	0.5	7	12
DF4	April 2020	0.5	6.5	11
DF5	April 2020	0.5	4	6
SY1	April 2020	0.5	7.5	13
SY2	April 2020	0.5	7	12
SY3	April 2020	0.5	6.5	11
BH1	April 2020	0.5	6.5	11
BH2	April 2020	0.5	8	14
BH3	April 2020	0.5	8	14
BH4	April 2020	0.5	8.5	15
BH5	April 2020	0.5	9	16
LYG1	April 2020	0.5	3	4
LYG2	April 2020	0.5	5.5	9
LYG3	April 2020	0.5	8.5	15
LYG4	April 2020	0.5	9	16
LYG5	April 2020	0.5	11	20
RD1	May 2020	0.5	9.05	16.1
RD2	May 2020	0.5	9.65	17.3
RD3	May 2020	0.5	4.35	6.7
RD4	May 2020	0.5	8.9	15.8
RD5	May 2020	0.5	14.1	26.2
DF1	May 2020	0.5	9.25	16.5
DF2	May 2020	0.5	5.5	9
DF3	May 2020	0.5	5	8
DF4	May 2020	0.5	7.5	13
DF5	May 2020	0.5	6.35	10.7
SY1	May 2020	0.5	7.5	13
SY2	May 2020	0.5	8.5	15
SY3	May 2020	0.5	9.5	17
BH1	May 2020	0.5	3.5	5
BH2	May 2020	0.5	8.75	15.5
BH3	May 2020	0.5	9	16
BH4	May 2020	0.5	9.25	16.5
BH5	May 2020	0.5	10	18
LYG1	May 2020	0.5	4	6
LYG2	May 2020	0.5	7	12
LYG3	May 2020	0.5	8.5	15
LYG4	May 2020	0.5	10	18
LYG5	May 2020	0.5	13	24
RD1	July 2020	0.5	10.75	19.5
RD2	July 2020	0.5	11	20
RD3	July 2020	0.5	5.5	9
RD4	July 2020	0.5	10	18
RD5	July 2020	0.5	14.75	27.5
DF1	July 2020	0.5	8	14
DF2	July 2020	0.5	9	16
DF3	July 2020	0.5	6	10
DF4	July 2020	0.5	5.75	9.5
DF5	July 2020	0.5	6.75	11.5
SY1	July 2020	0.5	8.25	14.5
SY2	July 2020	0.5	8	14
SY3	July 2020	0.5	8	14
BH1	July 2020	0.5	8.5	15
BH2	July 2020	0.5	9.25	16.5
BH3	July 2020	0.5	9.5	17
BH4	July 2020	0.5	9.5	17
BH5	July 2020	0.5	9.25	16.5
LYG1	July 2020	0.5	5.75	9.5
LYG2	July 2020	0.5	7.75	13.5
LYG3	July 2020	0.5	8	14
LYG4	July 2020	0.5	8.75	15.5
LYG5	July 2020	0.5	12	22

Note: Owing to wind- and current-induced vessel drift (0–500 m from target coordinates), actual sampling positions varied slightly across months, resulting in minor depth fluctuations at the same nominal station. Such positioning uncertainty is an inherent limitation of vessel-based oceanographic operations.

## Data Availability

Data are available from the corresponding authors upon reasonable request.

## References

[1] Sun Y., Yao L., Liu J., Tong Y., Xia J., Zhao X., Zhao S., Fu M., Zhuang M., He P. (2022). Prevention strategies for green tides at source in the Southern Yellow Sea. Mar. Pollut. Bull..

[2] Yu Z., Tang Y., Gobler C.J. (2023). Harmful algal blooms in China: History, recent expansion, current status, and future prospects. Harmful Algae.

[3] Liu J., Xia J., Zhuang M., Zhang J., Yu K., Zhao S., Sun Y., Tong Y., Xia L., Qin Y. (2021). Controlling the source of green tides in the Yellow Sea: NaClO treatment of *Ulva* attached on *Pyropia* aquaculture rafts. Aquaculture.

[4] Tong Y., Sun Y., Xia J., Liu J. (2025). Research progress on the characteristics of nitrogen and phosphorus uptake by *Ulva prolifera*, the dominant macroalga responsible for green tides in the Yellow Sea. Coasts.

[5] Li H., Zhang Y., Tang H., Shi X., Rivkin R.B., Legendre L. (2017). Spatiotemporal variations of inorganic nutrients along the Jiangsu coast, China, and the occurrence of macroalgal blooms (green tides) in the southern Yellow Sea. Harmful Algae.

[6] Li X., Gao H., Zhang C., Shi X. (2026). Multi-pathway nutrient replenishment mechanisms sustaining the transboundary green tides region in the Southern Yellow Sea. Mar. Environ. Res..

[7] Shan J., Li D., Li J., Hong L. (2026). From fighting alone to win-win cooperation: Horizontal ecological compensation in transboundary marine environmental governance. Ocean Coast. Manag..

[8] Yu T., Peng X.W., Wang Y., Xu S.W., Liang C., Wang Z. (2025). Green tide cover area monitoring and prediction based on multi-source remote sensing fusion. Mar. Pollut. Bull..

[9] Shao K., Gong N., Shen L., Han X., Wang Z., Zhou K., Kong D., Pan X., Cong P. (2024). Why did the world’s largest green tides occur exclusively in the Southern Yellow Sea?. Mar. Environ. Res..

[10] Huo Y., Zhang J., Chen L., Hu M., Yu K., Chen Q., He Q., He P. (2013). Green algae blooms caused by *Ulva prolifera* in the Southern Yellow Sea: Identification of the original bloom location and evaluation of biological processes occurring during the early northward floating period. Limnol. Oceanogr..

[11] Lamb A.L., Franklin D.J., Morris S., Sokolnicki J.R., Sulpice R., Fort A., Inabi M., Richier S., Le Bris A., Lemesle S. (2025). Macroalgal mat species diversity, composition, and seasonality at four coastal sites across the English Channel/La Manche region. Eur. J. Phycol..

[12] Taylor R., Fletcher R.L., Raven J.A. (2001). Preliminary studies on the growth of selected ‘Green tide’ algae in laboratory culture: Effects of irradiance, temperature, salinity and nutrients on growth rate. Bot. Mar..

[13] Han H., Zhao S., Song X., Wang H. (2023). The overwintering capability of *Ulva prolifera* spores and gametes in the Yellow Sea, China. J. Ocean Univ. China.

[14] Han H., Li Y., Ma X., Song W., Wang Z., Zhang X. (2022). Factors influencing the spatial and temporal distributions of green algae micro-propagules in the coastal waters of Jinmenghaiwan, Qinhuangdao, China. Mar. Pollut. Bull..

[15] Zhao J., Jiang P., Liu Z., Wei W., Lin H., Li F., Wang J., Qin S. (2013). The Yellow Sea green tides were dominated by one species, *Ulva* (*Enteromorpha*) *prolifera*, from 2007 to 2011. Chin. Sci. Bull..

[16] Hou W., Chen J., He M., Ren S., Fang L., Wang C., Jiang P., Wang W. (2024). Evolutionary trends and analysis of the driving factors of *Ulva prolifera* green tides: A study based on the random forest algorithm and multisource remote sensing images. Mar. Environ. Res..

[17] Ren C.-G., Liu Z.-Y., Zhong Z.-H., Wang X.-L., Qin S. (2022). Integrated biotechnology to mitigate green tides. Environ. Pollut..

[18] Li H.-M., Zhang C.-S., Han X.-R., Shi X.-Y. (2015). Changes in concentrations of oxygen, dissolved nitrogen, phosphate, and silicate in the Southern Yellow Sea, 1980–2012: Sources and seaward gradients. Estuar. Coast. Shelf Sci..

[19] Liu D., Keesing J.K., Xing Q., Shi P. (2009). World’s largest macroalgal bloom caused by expansion of seaweed aquaculture in China. Mar. Pollut. Bull..

[20] Wang Z., Fang Z., Liang J., Song X. (2023). Estimating *Ulva prolifera* green tides of the Yellow Sea through ConvLSTM Data Fusion. Environ. Pollut..

[21] Ma Y., Wong K., Tsou J.Y., Zhang Y. (2022). Investigating spatial distribution of green-tide in the Yellow Sea in 2021 using combined optical and SAR images. J. Mar. Sci. Eng..

[22] Tong Y., Tang P., Sun Y., Zhao S., Zhang J., Liu J., He P. (2022). Distribution characteristics of green algal micro-propagules in the East China Sea in winter and their relationship with green tide macroalgae in the Yellow Sea. J. Sea Res..

[23] Huo Y.Z., Hua L., Wu H.L., Zhang J.H., Cui J.J., Huang X.W., Yu K.F., Shi H.H., He P.M., Ding D.W. (2014). Abundance and distribution of *Ulva* microscopic propagules associated with a green tide in the southern coast of the Yellow Sea. Harmful Algae.

[24] Xing Q., Hu C. (2016). Mapping macroalgal blooms in the Yellow Sea and East China Sea using HJ-1 and Landsat data: Application of a virtual baseline reflectance height technique. Remote Sens. Environ..

[25] Bao M., Guan W., Yang Y., Cao Z., Chen Q. (2015). Drifting trajectories of green algae in the Western Yellow Sea during the spring and summer of 2012. Estuar. Coast. Shelf Sci..

[26] Zhang J., Huo Y., Wu H., Yu K., Kim J.K., Yarish C., Qin Y., Liu C., Xu R., He P. (2014). The origin of the *Ulva* macroalgal blooms in the Yellow Sea in 2013. Mar. Pollut. Bull..

[27] Li Q.Y., Yao A.L., Jiang X.T., Ming Y., Wang M.Y., Zhu L.X., Wang X.Y., Gao L. (2025). Distribution characteristics of nutrients in the Changjiang River Estuary under the watershed extreme drought in July 2023. Haiyang Xuebao.

[28] Ding L., Luan R. (2013). Flora Algarum Marinarum Sinicarum. Tomus IV, Chlorophyta. No.I, Ulotrichales Chaetophorales Phaeophilales Ulvales Prasiolales Cladophorales Acrosiphoniales.

[29] Pang S.J., Liu F., Shan T.F., Xu N., Zhang Z.H., Gao S.Q., Chopin T., Sun S. (2010). Tracking the algal origin of the *Ulva* bloom in the Yellow Sea by a combination of molecular, morphological and physiological analyses. Mar. Environ. Res..

[30] Liu F., Pang S., Chopin T., Gao S., Shan T., Zhao X., Li J. (2013). Understanding the recurrent large-scale green tide in the Yellow Sea: Temporal and spatial correlations between multiple geographical, aquacultural and biological factors. Mar. Environ. Res..

[31] Xia Z., Cao X., Li S., Cao J., Tong Y., Sun Y., Liu J., Zhao S., Cui Q., Zeng Y. (2023). Distribution of *Ulva prolifera*, the dominant species in green tides along the Jiangsu Province coast in the Southern Yellow Sea, China. J. Sea Res..

[32] Zhang X., Xu D., Mao Y., Li Y., Xue S., Zou J., Lian W., Liang C., Zhuang Z., Wang Q. (2011). Settlement of vegetative fragments of *Ulva prolifera* confirmed as an important seed source for succession of a large-scale green tide bloom. Limnol. Oceanogr..

[33] Shen Q., Li H., Li Y., Wang Z., Liu J., Yang W. (2012). Molecular identification of green algae from the rafts based infrastructure of *Porphyra yezoensis*. Mar. Pollut. Bull..

[34] Geng H., Yan T., Zhou M., Liu Q. (2015). Comparative study of the germination of *Ulva prolifera* gametes on various substrates. Estuar. Coast. Shelf Sci..

[35] Wang Z., Fan B., Yu D., Fan Y., An D., Pan S. (2022). Monitoring the spatio-temporal distribution of *Ulva prolifera* in the Yellow Sea (2020–2022) based on satellite remote sensing. Remote Sens..

[36] Wang S., Huo Y., Zhang J., Cui J., Wang Y., Yang L., Zhou Q., Lu Y., Yu K., He P. (2018). Variations of dominant free-floating *Ulva* species in the source area for the world’s largest macroalgal blooms, China: Differences of ecological tolerance. Harmful Algae.

[37] Wang Y., Liu F., Liu X., Shi S., Bi Y., Moejes F.W. (2019). Comparative transcriptome analysis of four co-occurring *Ulva* species for understanding the dominance of *Ulva prolifera* in the Yellow Sea green tides. J. Appl. Phycol..

[38] Han W., Chen L.-P., Zhang J.-H., Tian X.-L., Hua L., He Q., Huo Y.-Z., Yu K.-F., Shi D.-J., Ma J.-H. (2013). Seasonal variation of dominant free-floating and attached *Ulva* species in Rudong coastal area, China. Harmful Algae.

[39] Huan L., Gu W., Wang X., Yan Y., Tang Q., Han X., Wang Z., Zhou K., Qiu Q., Xu J. (2025). Reproductive traits of floating *Ulva prolifera* sporophytes and gametophytes and their contribution to the Yellow Sea green tide. Mar. Pollut. Bull..

[40] Yuan H., Xia J., Liu J., He P. (2025). “Love-hate relationships” between antibiotics and marine algae: A review. Aquat. Toxicol..

[41] Xia Z., Liu J., Zhao S., Sun Y., Cui Q., Wu L., Gao S., Zhang J., He P. (2024). Review of the development of the green tide and the process of control in the southern Yellow Sea in 2022. Estuar. Coast. Shelf Sci..

[42] Wang Y., Zhu D.K., Zhou L.F., Wang X.Y., Jiang S.L., Li H.Y., Shi B.W., Zhang Y.Z. (1998). Sediment characteristic and evolution of the radial tidal sandy ridges in the South Yellow Sea. Sci. Sin. Terrae.

[43] Kong X.H., Lu K., Xu X.D., Yang H.L., Zhang Y., Shang L.N. (2022). A study on the characteristic and its cause of topography and geomorphology of the South Yellow Sea. Mar. Geol. Quat. Geol..

[44] Zhang Y., He P., Li H., Li G., Liu J., Jiao F., Zhang J., Huo Y., Shi X., Su R. (2019). *Ulva prolifera* green-tide outbreaks and their environmental impact in the Yellow Sea, China. Natl. Sci. Rev..

[45] Ma X., Miao X., Fan S., Zang Y., Zhang B., Li M., Zhang X., Fu M., Wang Z., Xiao J. (2024). Dynamics of green macroalgal micro-propagules and the influencing factors in the Southern Yellow Sea, China. Sci. Total Environ..

[46] Song W., Jiang M., Wang Z., Wang H., Zhang X., Fu M. (2018). Source of propagules of the fouling green macroalgae in the Subei Shoal, China. Acta Oceanol. Sin..

[47] Li Y.-F., Geng H.-X., Hong X., Kong F.-Z., Yu R.-C. (2025). Strong interannual variation of green tides in the Southern Yellow Sea: Crucial factors and implications on management strategies. Mar. Pollut. Bull..

[48] Li Y.-F., Geng H.-X., Hong X., Kong F.-Z., Yu R.-C. (2020). Influence of iron and carbon on the occurrence of *Ulva prolifera* (Ulvophyceae) in the Yellow Sea. Reg. Stud. Mar. Sci..

[49] Li M., Miao X., Wang Y., Ma X., Zang Y., Liu X., Fan S., Zhang X., Wang Z., Xiao J. (2025). Succession of planktonic crustaceans responding to *Ulva* green tide in the Subei Shoal, Southwestern Yellow Sea. J. Ocean Univ. China.

[50] Sun J., Liu K., Zhang H., Fu J., Shi X., Yao Z., Zhao G., Sha Z., Cui H., Wu J. (2025). Dissipation of *Ulva prolifera* green tides across various spatial and temporal scales and the short-term effects on marine environments. Mar. Environ. Res..

[51] Fan L., Chen Y., Song X., Zhao J., Shi X., Li K., Wang X. (2026). The regulatory effect of environmental factors on green tide blooms in the Yellow Sea: A spatio-temporal downscaling numerical reconstruction method. Mar. Environ. Res..

[52] Chen X., Yu Z., Fu Y., Dong M., Zhang J., Yao Q. (2024). Seasonal and interannual variations of nutrients in the Subei Shoal and their implication for the world’s largest green tide. Sci. Total Environ..

[53] Tang P., Du P., Guo S., Qie L., Zhang W., Zhang P., Réus M., Chanussot J. (2026). A fully automatic and label-free sentinel-1 SAR framework for green-tide mapping. Int. J. Appl. Earth Obs. Geoinf..

[54] Yin Z., Tang J., Lu Y., Liu Y., Duan H., Jiao J., Xing Q., Li J., Liu Y. (2025). Characterizing distribution patterns of small algae patches across the northern and southern sides of the Yellow Sea front using synchronous CZI-MODIS images. Int. J. Remote Sens..

[55] Zhu W., Xu Y., Zhang L., Liu Z., Liu S., Li Y. (2025). A deep-learning framework to detect green tide from MODIS images. Front. Remote Sens..

[56] Ding Y., Gao S., Huang G., Wu L., Wang Z., Yuan C., Yu Z. (2024). A novel method for simplifying the distribution envelope of green tide for fast drift prediction in the Yellow Sea, China. Remote Sens..

[57] Zhang G., He Y., Niu L., Wu M., Kaufmann H., Liu J., Liu T., Kong Q., Chen B. (2024). Identification of green tide decomposition regions in the Yellow Sea, China: Based on time-series remote sensing data. Remote Sens..

[58] Li S., Xia Z., Cao J., Zhang J., He P. (2024). Distribution of *Ulva* macroalgae before and after the outbreak of the Yellow Sea green tide in aquaculture ponds and rivers nearshore Jiangsu, China. Reg. Stud. Mar. Sci..

[59] Cao J., Zeng Y., Xia Z., Li S., He P., Zhang J. (2024). Spatio-temporal distribution of micropropagules of green algae along the Jiangsu coast. Mar. Environ. Res..

[60] Xia Z., Yuan H., Liu J., Zhao S., Tong Y., Sun Y., Li S., Li A., Cao J., Xia J. (2022). Biomass and species composition of green macroalgae in the Binhai Harbor intertidal zone of the southern Yellow Sea. Mar. Pollut. Bull..

[61] Xia Z., Liu J., Zhao S., Cui Q., Bi F., Zhang J., He P. (2023). Attached *Ulva meridionalis* on nearshore dikes may pose a new ecological risk in the Yellow Sea. Environ. Pollut..

[62] Huo Y., Han H., Shi H., Wu H., Zhang J., Yu K., Xu R., Liu C., Zhang Z., Liu K. (2015). Changes to the biomass and species composition of *Ulva* sp. on *Porphyra* aquaculture rafts, along the coastal radial sandbank of the Southern Yellow Sea. Mar. Pollut. Bull..

[63] Li Y., Song W., Xiao J., Wang Z., Fu M., Zhu M., Li R., Zhang X., Wang X. (2014). Tempo-spatial distribution and species diversity of green algae micro-propagules in the Yellow Sea during the large-scale green tide development. Harmful Algae.

[64] Yang J., Yin Y., Yu D., He L., Shen S. (2021). Activation of MAPK signaling in response to nitrogen deficiency in *Ulva prolifera* (Chlorophyta). Algal Res..

[65] He Y., Ao Y., Yin Y., Yuan A., Che T., Li L., Shen S. (2019). Comparative transcriptome analysis between floating and attached *Ulva prolifera* in studying green tides in the Yellow Sea. Algal Res..

[66] Zhao H., Liu X., Jiang T., Cai C., Gu K., Liu Y., He P. (2022). Activated abscisic acid pathway and C_4_ pathway, inhibited cell cycle progression, responses of *Ulva prolifera* to short term high temperature elucidated by multi-omics. Mar. Environ. Res..

[67] Gu K., Liu Y., Jiang T., Cai C., Zhao H., Liu X., He P. (2022). Molecular response of *Ulva prolifera* to short-term high light stress revealed by a multi-omics approach. Biology.

[68] He H., Yang J., He Y., Yang X., Fu C., Zhang D., Dong J., Zeb A., Qu J., Shen S. (2024). Proliferating cell nuclear antigen of *Ulva prolifera* is involved in the response to temperature stress. J. Oceanol. Limnol..

[69] Zeng Y., Chen Z., Cao J., Li S., Xia Z., Sun Y., Zhang J., He P. (2024). Revolutionizing early-stage green tide monitoring: eDNA metabarcoding insights into *Ulva prolifera* and microecology in the South Yellow Sea. Sci. Total Environ..

[70] Stoeckle B.C., Kuehn R., Geist J. (2016). Environmental DNA as a monitoring tool for the endangered freshwater pearl mussel (*Margaritifera margaritifera* L.): A substitute for classical monitoring approaches?. Aquat. Conserv. Mar. Freshw. Ecosyst..

[71] Xing Y., Gao W., Shen Z., Zhang Y., Bai J., Cai X., Ouyang J., Zhao Y. (2022). A review of environmental DNA field and laboratory protocols applied in fish ecology and environmental health. Front. Environ. Sci..

[72] Buxton A., Matechou E., Griffin J., Diana A., Griffiths R.A. (2021). Optimising sampling and analysis protocols in environmental DNA studies. Sci. Rep..

[73] Dimond J.L., Gathright B.R., Bouma J.V., Carson H.S., Sowul K. (2022). Detecting endangered pinto abalone (*Haliotis kamtschatkana*) using environmental DNA: Comparison of ddPCR, qPCR, and conventional diver surveys. Environ. DNA.

[74] Burian A., Mauvisseau Q., Bulling M., Domisch S., Qian S., Sweet M. (2021). Improving the reliability of eDNA data interpretation. Mol. Ecol. Resour..

[75] Allan E.A., Shaffer M.R., Kelly R.P., Parsons K. (2025). Optimizing target-to-total DNA ratio in eDNA studies: Effects of sampling, preservation, and extraction methods on single-species detection. PeerJ.

[76] Majaneva M., Diserud O.H., Eagle S.H.C., Boström E., Hajibabaei M., Ekrem T. (2018). Environmental DNA filtration techniques affect recovered biodiversity. Sci. Rep..

[77] Bessey C., Jarman S.N., Simpson T., Miller H., Stewart T., Keesing J.K., Berry O. (2021). Passive eDNA collection enhances aquatic biodiversity analysis. Commun. Biol..

[78] Schabacker J.C., Amish S.J., Ellis B.K., Gardner B., Miller D.L., Rutledge E.A., Sepulveda A.J., Luikart G. (2020). Increased eDNA detection sensitivity using a novel high-volume water sampling method. Environ. DNA.

[79] George S.D., Sepulveda A.J., Hutchins P.R., Pilliod D.S., Klymus K.E., Thomas A.C., Augustine B.C., Adrianza C.C.H., Jones D.N., Williams J.R. (2024). Field trials of an autonomous eDNA sampler in lotic waters. Environ. Sci. Technol..

[80] Liu Q., Tan J., Wang M., Xin N., Qi R., Wang H. (2024). Optimization of pore size and filter material for better enrichment of environmental DNA. Front. Environ. Sci..

[81] Veríssimo J., Lopes-Lima M., Amaral F., Chaves C., Fernandes V., Kemanja M., Teixeira A., Martins F.M.S., Beja P. (2025). Navigating methodological trade-offs in edna metabarcoding biodiversity monitoring: Insights from a Mediterranean watershed. Mol. Ecol. Resour..

[82] Mei L., How C.M., Sun M., Yan R., Zheng W., Zhang Y., Hu H., Huang B., Qiu J.-W., Zeng Z. (2026). A tailored MoS_2_ membrane with strong DNA binding capability enhances aquatic biota detection through environmental DNA metabarcoding. Natl. Sci. Rev..

